# Multisystem Immune-Related Adverse Events Following Pembrolizumab: A Critical Care Case of Myocarditis, Diabetic Ketoacidosis, and CNS Demyelination

**DOI:** 10.7759/cureus.90424

**Published:** 2025-08-18

**Authors:** Tomas Janulevicius, James Nguyen, Edward Briggs

**Affiliations:** 1 Department of Intensive Care, Joondalup Health Campus, Perth, AUS

**Keywords:** immune checkpoint inhibitor, immune checkpoint inhibitor adverse effects, immune mediated demyelination, immune-related adverse event (irae), immune therapy-mediated myocarditis, immunotherapy induced diabetes, immunotherapy induced dka, immunotherapy-related adverse events

## Abstract

The advent of immune checkpoint inhibitors has significantly advanced cancer treatment. Pembrolizumab, an immune checkpoint inhibitor, has been shown to significantly improve both the progression-free interval and overall survival of patients with melanoma. However, the use of these medications is not without drawbacks and can lead to immune-related adverse events (irAEs), which, though rare, can have permanent sequelae.

We present a case of a male in his fifties with a history of melanoma who developed severe multi-system dysfunction following pembrolizumab therapy. Five days following his third cycle of pembrolizumab, the patient presented in severe diabetic ketoacidosis, with hemodynamic instability and acute kidney injury. Subsequently, he developed myocarditis and central nervous system (CNS) demyelination. Management involved high-dose corticosteroids, empiric antimicrobial therapy, and supportive measures including invasive ventilation, hemodynamic support, and renal replacement therapy.

This case illustrates the complex and evolving spectrum of pembrolizumab-related immune toxicity and contributes to the literature by documenting a rare combination of multisystem irAEs occurring in close succession. It reinforces the importance of early recognition and prompt, tailored management of irAEs in patients receiving immune checkpoint inhibitors.

## Introduction

Immune checkpoint inhibitors (ICIs), such as pembrolizumab, are an evolving class of drugs that have changed the field of immuno-oncology. Historically, the five-year survival for patients with metastatic melanoma was approximately 10% [1]. With the advent of ICIs, studies have shown significant improvement in both progression-free and overall survival [2,3].

Pembrolizumab is a monoclonal antibody that targets the PD-1 receptor on activated T-cells. In the normal physiological state, PD-L1 on cell surfaces binds to PD-1 receptors on lymphocytes to inhibit cytokine secretion and induce apoptosis. Through this mechanism, the PD-1/PD-L1 pathway mediates immune tolerance and provides immune homeostasis [4]. By blocking this interaction, pembrolizumab not only upregulates immune response against tumour cells but also increases the risk of immune-related adverse events (irAEs). IrAEs occur in approximately 1 in 4 (26.82%) of individuals treated with ICIs, with 6.1% experiencing severe adverse events [5]. The most affected systems include the dermatological, musculoskeletal, and pulmonary systems, though rarer endocrine, neurological, and cardiovascular irAEs are documented [6].

This case report details the progress of a patient who developed life-threatening multiorgan dysfunction following treatment with pembrolizumab, and contributes to the growing body of evidence of irAEs associated with ICIs.

## Case presentation

A male in his fifties, with a background of dyslipidaemia and resected stage IIIA melanoma (BRAF/NRAS/KIT wild-type), presented to the emergency department with a two-day history of nausea, vomiting (>20 episodes/day), diarrhoea, and increased work of breathing. He denied hematemesis, abdominal pain, fevers, or rigors. Notably, the patient had received his third 200 mg dose of pembrolizumab intravenously five days prior to presentation as adjuvant melanoma therapy.

Initial evaluation

The patient presented in shock with cyanotic peripheries and Kussmaul respirations. Examination findings revealed tachypnoea, tachycardia, and hypotension with a mean arterial pressure (MAP) of 61 mmHg (Table 1). His Glasgow Coma Scale (GCS) was 13/15 (E4/V4/M5). The respiratory, cardiovascular, abdominal, and neurological examinations were otherwise unremarkable. The patient was profoundly diaphoretic but apyrexial.

**Table 1:** Initial examination findings

System	Examination Findings
Respiratory	Respiratory rate 30. Oxygen saturation 98%, Room air. Kussmaul respirations. No added breath sounds. No focal crepitations.
Cardiovascular	Heart rate 111. Blood pressure 77/44 mmHg, mean arterial pressure 61 mmHg. Dual heart sounds, no murmur. Cool peripheries, cyanotic.
Abdominal	Soft, non-tender. Bowel sounds present.
Neurological	Glasgow coma scale 13/15 (E4/V4/M5). Pupils equal and reactive. Kernig’s/Bruzinski’s sign negative. No neck stiffness. No photophobia.

Initial blood testing revealed severe metabolic acidosis (pH 6.99, HCO_3_- 4.3 mmol/L), severe hyperglycemia (glucose 58 mmol/L), elevated capillary ketones (7.3 mmol/L), and elevated serum osmolality (358 mmol/kg).

White cell count (12.6 x10^9^/L), C-reactive protein (257 mg/L), and lactate (2.9 mmol/L) were elevated suggesting an infective or inflammatory process, and the renal profile revealed a stage three acute kidney injury (AKI; creatinine 456 µmol/L), hyperosmolar hyponatremia (serum sodium 106 mmol/L; 127 mmol/L when corrected for hyperglycemia), and hyperkalemia (potassium 6.2 mmol/L). Liver function tests (LFTs) were unremarkable, but lipase was elevated (659 IU/L). CT-abdomen/pelvis showed no evidence of pancreatitis or other focal pathology. A summary of laboratory investigations is included in Table 2.

**Table 2:** Admission laboratory investigations demonstrating severe metabolic, renal, and endocrine abnormalities Severe metabolic acidosis with markedly elevated inflammatory markers is evident, suggestive of an underlying infective or inflammatory process. The presence of high capillary ketones, hyperglycaemia, and reduced bicarbonate supports a diagnosis of diabetic ketoacidosis. Renal function tests indicate acute kidney injury, with hyperkalemia likely secondary to acidemia. Liver function tests are within normal limits, and elevated serum cortisol suggests an appropriate stress response. Abbreviations: pH: potential of hydrogen, pCO_2_: partial pressure of carbon dioxide, HCO_3_^-^: bicarbonate, Na^+^: sodium, K^+^: potassium, Cl^-^: chloride, Hb: hemoglobin, WCC: white cell count, ALP: alkaline phosphatase, GGT: gamma-glutamyl transferase, ALT: alanine aminotransferase

Investigation		Patient Values	Reference Range
Venous Blood Gas	pH	6.99	7.35 – 7.45
	pCO_2_	18.7 mmHg	36 – 46 mmHg
	HCO_3_^-^	4.3 mmol/L	22 – 26 mmol/L
	Na^+^	116 mmol/L	135 – 145 mmol/L
	K^+^	7.2 mmol/L	3.5 – 5.2 mmol/L
	Cl^-^	78 mmol/L	95 – 110 mmol/L
	Glucose	58 mmol/L	4 – 10 mmol/L
	Lactate	2.9 mmol/L	<1.3 mmol/L
	Creatinine	438 µmol/L	60 – 110 µmol/L
Full Blood Count	Hb	140 g/L	133 – 170 g/L
	Platelets	92 x10^9^/L	150 – 450 x10^9^/L
	WCC	12.6 x10^9^/L	4.0 – 11.0 x10^9^/L
Renal Profile	Creatinine	456 µmol/L	60 – 110 µmol/L
	Urea	28 mmol/L	3.0 – 8.0 mmol/L
	HCO_3_^-^	4.0 mmol/L	22 – 26 mmol/L
	Cl^-^	69 mmol/L	95 – 110 mmol/L
	K^+^	6.2 mmol/L	3.5 – 5.2 mmol/L
	Na^+^	106 mmol/L	135 – 145 mmol/L
Liver Function Tests	Bilirubin	9 µmol/L	<21 µmol/L
	ALP	99 U/L	30 – 110 U/L
	GGT	43 U/L	<51 U/L
	ALT	36 U/L	<56 U/L
	Albumin	40 g/L	38 – 50 g/L
	Total protein	60 g/l	60 – 80 g/L
Other Tests	Capillary Ketones	7.3 mmol/L	<0.6 mmol/L
	C-Reactive Protein	257 mg/L	<5 mg/L
	Serum Osmolality	358 mmol/kg	275-295 mmol/kg
	Creatine Kinase	3575 U/L	<200 U/L
	Lipase	659 IU/L	13 – 60 IU/L
	Cortisol	2120 nmol/L	185 – 624 nmol/L

**Figure 1:**
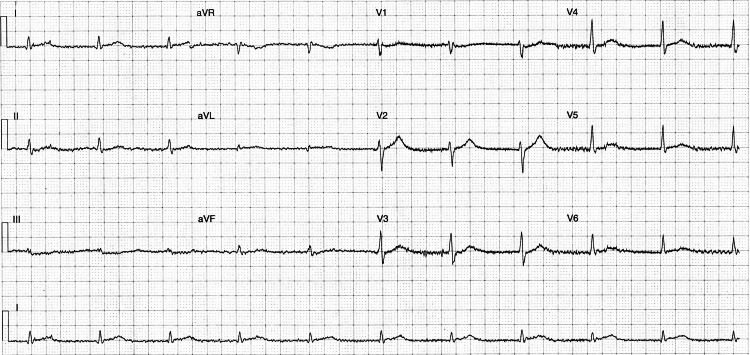
Initial 12-lead electrocardiogram Junctional escape rhythm characterized by a lack of discernible P waves while maintaining a regular pattern with a narrow QRS complex

Initial electrocardiogram (ECG) showed an intermittent junctional escape rhythm (Figure 1), and high-sensitivity troponins were elevated at 238 µg/L (hsTnI; Beckman Coulter, California, US).

**Figure 2:**
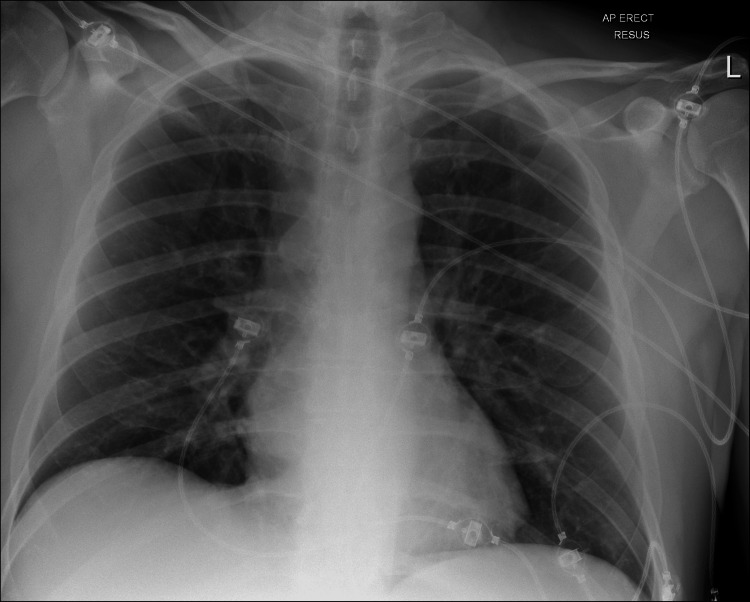
Initial chest X-ray Anterior-posterior erect view

Plain chest radiograph (Figure 2) and CT-brain were unremarkable.

Urine dipstick showed glycosuria and microscopic hematuria with no leucocytes or nitrites, and an extended viral respiratory panel was negative.

Additional testing, including C-peptide, islet autoantibodies (Anti-GAD65/Anti-IA2), blood cultures, procalcitonin, and an extended autoimmune panel, was sent on admission, but results were not available during the acute management phase.

Differential diagnosis

The list of issues and differentials at this point was broad. The primary considerations are shown in Table 3.

**Table 3:** Summary of initial clinical issues and differential diagnoses Abbreviations: DKA, diabetic ketoacidosis; HHS, hyperosmolar hyperglycaemic state

Issue	Differential Diagnosis
Shock	Most likely precipitated by hypovolaemia and severe DKA. A systemic inflammatory response was also noted, but without any localizing signs of an underlying focus of sepsis.
Metabolic/Endocrine	Mixed severe DKA-HHS overlap was considered, despite no prior history of diabetes mellitus (DM); in the context of extremely low bicarbonate and high ketones, a primarily DKA-type picture was deemed most likely, with the elevated lipase attributed to possible immune-related pancreatic injury, given the absence of CT evidence of pancreatitis.
Cardiac	Cardiac involvement was evident due to elevated troponins and an intermittent junctional escape rhythm. This was attributed to a type-two myocardial infarction secondary to shock; however, immune-related myocarditis and acute coronary syndrome remained part of the differential diagnosis.
Renal	Acute kidney injury was presumed to be pre-renal in the context of hypovolemic shock, with preserved urine output suggesting a degree of maintained renal perfusion. Nephritis remained a differential diagnosis.

An irAE secondary to pembrolizumab was a key differential and offered a potential unifying diagnosis, but sepsis was also considered and treated. The decision was made to stabilize the patient’s metabolic state before commencing corticosteroid therapy. Furthermore, no definitive life-threatening irAE was yet identified, necessitating immediate steroid therapy. This approach was in keeping with emerging evidence suggesting that corticosteroids do not provide therapeutic benefit in ICI-induced diabetic ketoacidosis (DKA) [7].

Management and organ support

Following initial assessment, aggressive fluid resuscitation (3 litres of crystalloid over an hour), intravenous insulin infusion (commenced at 10 units/hour, titrated), and empiric antimicrobial therapy (piperacillin-tazobactam 4.5d t.d.s.) were initiated. 

Initially, peripheral intravenous metaraminol was commenced for persistent hypotension, titrated to maintain a MAP of >65 mmHg before conversion to noradrenaline due to worsening hypotension. Due to worsening agitation and shock, the patient was intubated with consideration to mitigate acidosis through controlled hyperventilation.

While acidaemia, ketosis, and hyperglycaemia began to improve within hours of initiating standard DKA management, the patient developed oligo-anuric renal failure despite ongoing fluid resuscitation and vasopressor support. A dialysis catheter was inserted, and renal replacement therapy (RRT) was initiated using modified dialysate to avoid rapid sodium correction. 

**Figure 3:**
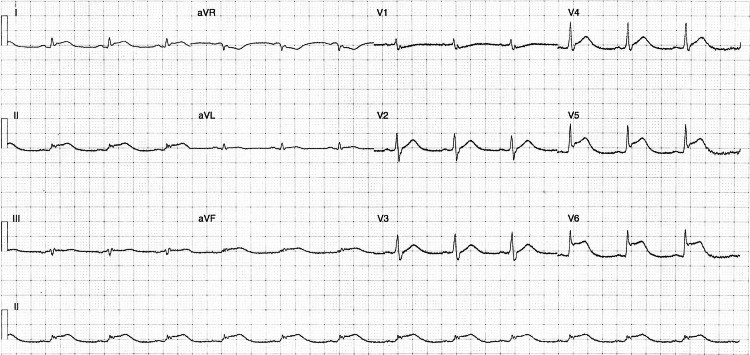
Follow-up 12-lead electrocardiogram Infero-lateral ST-elevation with no reciprocal ST-depression. No T-wave inversion. RSR’ pattern in V1. Narrow QRS complex. This focal ST-elevation pattern is suggestive of a focal occlusion in a coronary artery.

**Figure 4:**
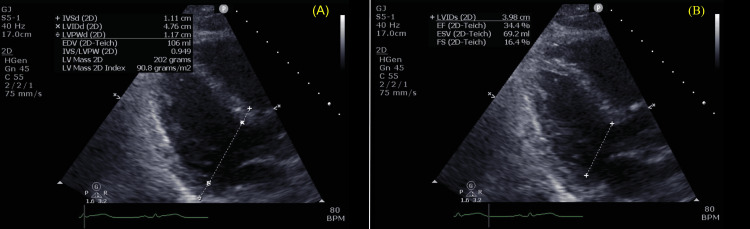
Transthoracic echocardiogram demonstrating systolic dysfunction (A) Modified four-chamber view at end-diastole. (B) Four-chamber view at end-systole. Teichholz-derived measurements demonstrate an impaired left ventricular ejection fraction (LVEF) of 34% and right ventricular dilation. These findings are consistent with systolic dysfunction and, in the clinical context of elevated troponin and ST-segment elevation, raise suspicion for acute coronary syndrome with a differential diagnosis of immune-related myocarditis. Abbreviations: IVSd, interventricular septum thickness end-diastole; LVIDd, left ventricular internal diameter end-diastole; LVPWd, left ventricular posterior wall thickness end-diastole; EDV, end-diastolic volume; LV, left ventricle; LVIDs, left ventricular internal diameter end-systole; EF, ejection fraction; ESV, end-systolic volume; FS, fractional shortening.

On day two, ECG monitoring demonstrated regional infero-lateral ST-elevation (Figure 3) and raised serial serum troponins from 238 µg/L to 34,923 µg/L. Transthoracic echocardiography showed severely impaired systolic function with a left ventricular ejection fraction (LVEF) of 34% (Figure 4).

**Figure 5:**
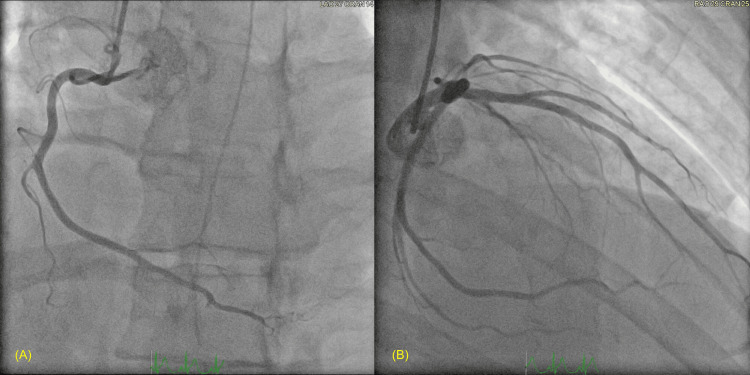
Coronary angiography showing non-obstructive disease (A) Left anterior oblique (LAO 27°) cranial (14°) view. (B) Right anterior oblique (RAO 29°) cranial (25°) view. Mild irregularities are seen in the left anterior descending artery without occlusive coronary plaque. These findings rule out acute coronary syndrome as a cause of the elevated troponins, making immune-related myocarditis the most likely differential.

The patient received dual antiplatelet loading, and an urgent coronary angiogram was performed, which revealed no obstructing coronary artery disease (Figure 5). In light of this, intravenous methylprednisolone was commenced at a dose of 250 mg o.d. for presumed ICI-myocarditis (Grade 4, Common Terminology Criteria for Adverse Events (CTCAE)).

Over the following 48 hours, metabolic parameters improved and cardiac function normalized. RRT and cardiorespiratory support were successfully weaned, and the patient was extubated.

Admission blood cultures subsequently grew Staphylococcus lugdunensis and Staphylococcus capitis, and procalcitonin was elevated at 1.95 μg/L. The clinical relevance of these findings is explored further in the discussion section.

Neurological complication

**Figure 6:**
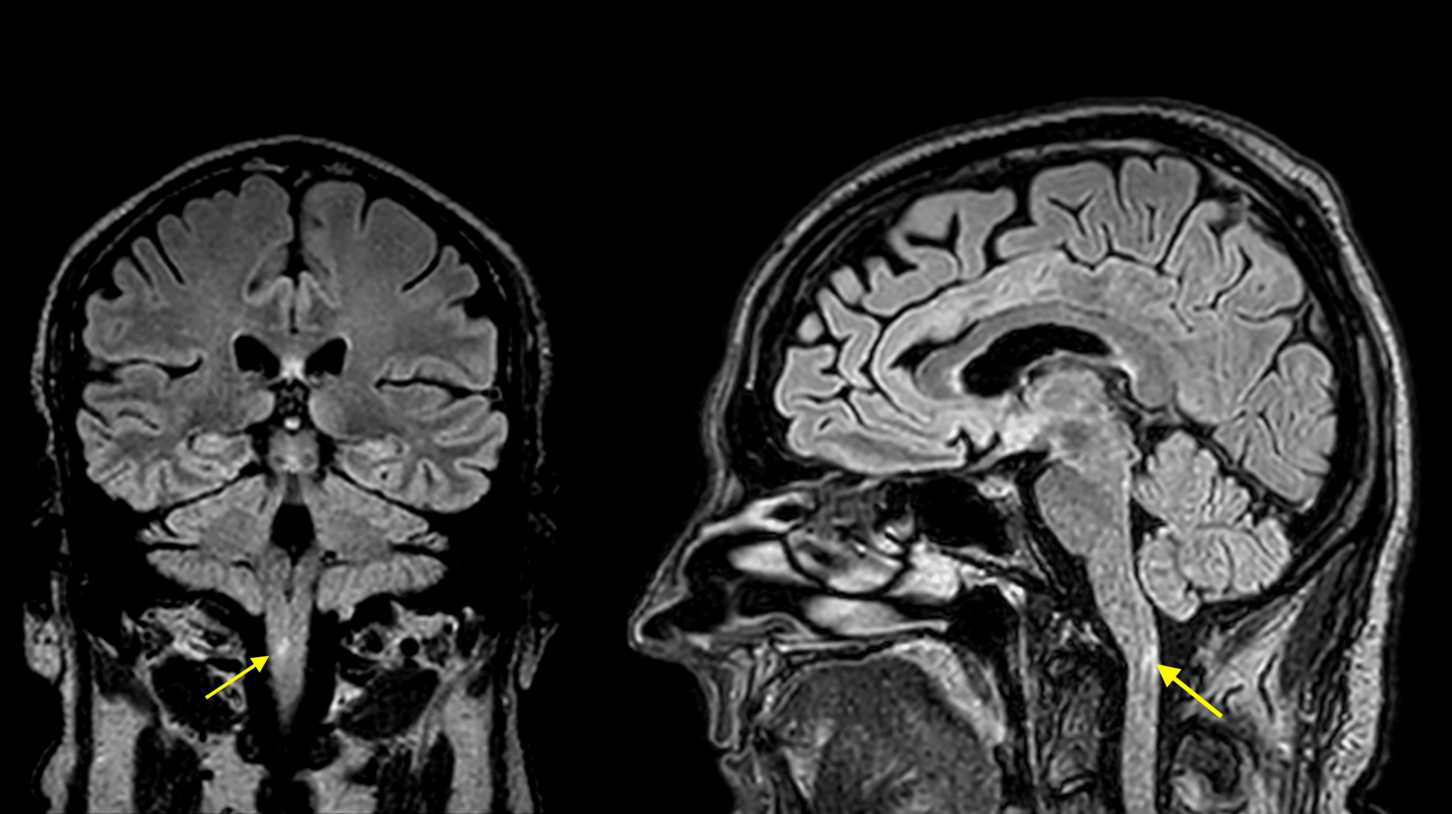
FLAIR MRI demonstrating demyelination in the area postrema Coronal and sagittal fluid attenuated inversion recovery (FLAIR) MRI views showing a hyperintense lesion in the caudal medulla (yellow arrows), consistent with demyelination of the area postrema. This circumventricular organ acts as a chemoreceptor trigger zone for emesis by detecting circulating toxins. Its involvement likely explains the patient’s persistent vomiting.

Despite overall improvement with immunosuppression, new-onset neurological symptoms were noted shortly after extubation. The patient developed intractable nausea, hyperemesis, and hiccups refractory to antiemetic treatment. Regular ondansetron, cyclizine, midazolam, and droperidol PRN were trialled with minimal effect. Neurological examination revealed bilateral clonus, hyperreflexia in lower limbs, and ataxic gait. MRI brain revealed a focal area of T2/fluid attenuated inversion recovery (FLAIR) hyperintensity in the right posterior paramedian aspect of the cervicomedullary junction with subtle FLAIR hyperintensity in the area postrema (Figure 6), consistent with a central demyelinating process at the chemoreceptor trigger zone.

Given the favourable clinical response to corticosteroids, MRI findings that were typical of an immune-related demyelinating process, and the absence of clinical features concerning for CNS infection, a conservative monitoring approach was adopted. A lumbar puncture was deemed unnecessary at the time based on clinical judgment.

Outcome and follow-up

On day nine of admission, the patient was discharged from the ICU to the ward. ECGs showed resolution of ST-segment changes, and a repeat echocardiogram showed return of normal left ventricular function. The central vomiting syndrome and neurological deficits showed complete resolution, and the patient was discharged from the hospital 11 days following presentation.

Following resolution of the acute metabolic disturbance, the patient remained an insulin-dependent diabetic and on a subcutaneous insulin regimen. The patient’s renal function continued to improve, with creatinine stabilizing at 98 µmol/L when followed up in the outpatient clinic three months later. 

The severe irAEs following pembrolizumab therapy precluded further immunotherapy for the patient. However, at oncological follow-up, with a positron emission tomography (PET) scan three months post-discharge, the patient remained disease-free.

## Discussion

Case overview

We describe a challenging and clinically evolving case of life-threatening pembrolizumab-related toxicity affecting multiple organ systems, including fulminant DKA, myocarditis, and a focal neuroinflammatory process. Although each of these complications is rare, reported in close to 1% for DKA [8], 0.32% (3.2 per 1,000) for myocarditis [9], and 0.5% for central nervous system involvement [10], the simultaneous occurrence in a single individual is exceedingly rare and scarcely documented in existing literature.

This case underscores the challenges of recognising irAEs early when the presenting symptoms overlap with more commonly seen differentials such as sepsis or acute coronary syndrome. The clinical course required frequent reconsideration of the leading differentials and adaptation of management strategies, such as manually diluting dialysate bags and careful risk-benefit analysis of immunosuppression, in the setting of severe metabolic disturbance and potentially undifferentiated sepsis. 

While corticosteroids remain a cornerstone of irAE treatment, this case reinforces that the timing and appropriateness of their use is often nuanced and context-dependent. In this case, early corticosteroid use may have worsened the patient’s metabolic state, while delayed treatment risked further immune-mediated injury. Ultimately, the clinical decision-making process involved addressing immediately life-threatening abnormalities while maintaining a broad differential and anticipating complications. Following the commencement of corticosteroids, clinical improvement was not immediate, highlighting the need for robust supportive care alongside immunosuppression.

Concurrent sepsis

Sepsis remained a key differential at presentation, and empiric broad-spectrum antibiotics were commenced. Blood cultures subsequently grew *Staphylococcus (S.) capitis* (often a contaminant [11]) and *S. lugdunensis* (clinically significant [12]). Despite no clear source identified, the elevated procalcitonin (1.95 μg/L), and the severity of illness prompted treatment of *S. lugdunensis* as a true pathogen. 

While the septic process likely contributed to the degree of shock and requirement for critical intervention on presentation, the bacteremia was likely a secondary bloodstream infection secondary to prolonged hyperglycaemia and immunosuppression, rather than a unifying diagnosis. Nonetheless, the concurrent sepsis complicated the decision-making process and reinforced the challenge in balancing early immunosuppression with adequate antimicrobial coverage.

Endocrine toxicity: ICI-induced DKA

The patient presented with new-onset DKA (Grade 4, CTCAE) without a prior history of DM or insulin resistance. In light of recent pembrolizumab therapy and emerging evidence of its association with DM, ICI-induced diabetes mellitus (ICI-DM) was a key differential. Other key differentials were latent autoimmune diabetes in adults (LADA) and pancreatitis-induced DKA (lipase 657 IU/L), though pancreatitis was ruled out, as examination and radiological imaging were normal. 

Blood tests revealed weakly positive anti-GAD65 antibodies (6.6 IU/ml), negative anti-IA2 antibodies (5.2 IU/ml), elevated HbA1c (8.3%), and low but detectable C-peptide (0.15 nmol/L). This weak anti-GAD65 positivity, elevated HbA1c, and low C-peptide level support an ICI-induced LADA phenotype rather than fulminant ICI-DM, which typically presents with undetectable C-peptide levels and abrupt onset [13]. The proposed mechanism in our patient is an ICI-triggered activation of an autoimmune predisposition to DM, which is supported by emerging literature [14].

Unlike other irAEs, expert consensus and emerging guidelines state there is no role for corticosteroids in the management of ICI-induced DKA, and the use of corticosteroids may worsen insulin resistance [7].

Cardiac toxicity: ICI-myocarditis

On presentation, the patient’s ECG showed a junctional escape rhythm at 60 bpm. This was initially attributed to a combination of hyperkalemia (6.2 mmol/L), acidemia (pH 6.99), and critical illness in the context of severe DKA. Equally, a troponin of 238 µg/L was contextually equivocal due to the degree of shock demonstrated. As the case evolved, these factors later became potential early indicators of cardiac involvement. This is consistent with emerging data, with arrhythmias reported in close to 40% of patients with ICI-myocarditis, including both supraventricular (23.8%) and life-threatening ventricular arrhythmias (15%) [15].

The patient subsequently developed regional ECG changes (infero-lateral ST-elevation), elevated troponins (34,923 µg/L), and impaired systolic function (LVEF 34%), posing a major diagnostic dilemma. While ICI-myocarditis was a key differential, the presence of regional ECG changes, dyslipidaemia, and echocardiogram findings raised the possibility of myocardial infarction. Given concern for acute coronary syndrome and possible sepsis, coronary angiography was prioritised before initiating high-dose corticosteroids. This reflected the concerns that in the absence of a clear and compelling diagnosis of ICI-myocarditis, high-dose corticosteroids may suppress the host immune response and subsequently increase infection-related mortality risk. Angiography revealed no significant coronary artery disease, strongly supporting a diagnosis of focal myocarditis and prompting initiation of high-dose corticosteroids following immunology consultation.

This evolving picture likely reflected progressive inflammation from a focal origin, a pattern supported by literature describing patchy myocardial involvement in ICI-myocarditis. While biopsy remains the gold standard for diagnosis, its invasive nature and the often nonuniform distribution of myocardial involvement limit its real-world usage. Cardiac magnetic resonance (CMR) is a promising non-invasive alternative [16], but its high false negative rate underscores the challenge of relying on a single modality for diagnosis [17]. 

This case highlights how early clinical suspicion, even in the absence of definitive imaging, remains central to guiding treatment. High-dose corticosteroids remain the first-line treatment for ICI-myocarditis, with emerging therapies, such as anti-thymocyte globulin (ATG), explored in severe or refractory cases [18].

Neurological irAEs and area postrema syndrome

Following extubation, the patient experienced intractable nausea, vomiting, and hiccups unresponsive to antiemetic therapy. Neurological examination revealed bilateral lower limb clonus, hyperreflexia, and ataxia. MRI demonstrated focal T2/FLAIR hyperintensity in the right posterior paramedian cervicomedullary junction and area postrema. These findings aligned with the patient’s clinical signs: interruption of descending corticospinal fibres explained the upper motor neuron features, while involvement of the dorsal column-medial lemniscus system accounted for the sensory ataxia. Involvement of the area postrema (chemoreceptor trigger zone) accounted for the vomiting syndrome and raised suspicion for neuromyelitis optica spectrum disorder (NMOSD)-like demyelination. NMOSD-like demyelination is becoming increasingly recognised as a rare irAE of ICI therapy [19].

Key differentials included osmotic demyelination syndrome (ODS), meningoencephalitis, and CNS metastases. ODS was thought unlikely due to the lesion location, dilution of dialysate on RRT, and absence of characteristic pontine involvement. While bacteraemia was present, these organisms were not typically neuroinvasive, and there were no clinical or radiological signs of CNS infection. Micro-metastases could not be fully excluded, though a normal follow-up MRI at three months made this less likely.

Neurological irAEs are reported less frequently and typically manifest later than other irAEs. Their response to corticosteroids is typically favourable, with relapse being uncommon [20]. In this case, the timing, focal findings, and steroid responsiveness strongly support a diagnosis of ICI-associated CNS demyelination, classified as Grade 3 under CTCAE. Given the classic presentation and response to therapy, specific NMOSD antibody testing was not pursued at the time. While clinically justified in this context, this remains a limitation, and future cases of ICI-associated demyelination should include such testing.

## Conclusions

We have detailed a patient who developed a rare and clinically complex combination of severe irAEs--DKA, myocarditis, and CNS demyelination--following pembrolizumab therapy. The initial presentation was characterized by shock and severe metabolic disturbance, appropriately raising concerns for more common differentials like sepsis and acute coronary syndrome. As the case unfolded, new findings required consistent re-evaluation of the working diagnoses, and a balancing of the risk of high-dose corticosteroids, especially in the context of undifferentiated shock and severe DKA.

The subsequent development of neurological symptoms not only further complicated the clinical picture but also highlighted how irAEs can evolve sequentially, resemble more familiar pathology, and vary in responsiveness to treatment. Despite the extent of multiorgan involvement, the patient made a near-complete recovery. This case underscores the real-world complexity of ICI therapy and the importance of early multidisciplinary team input, judicious use of immunosuppression, and robust supportive care. Furthermore, this case adds to a growing body of evidence of irAEs associated with ICI therapy.
